# Padrões de refluxo nas veias safenas em homens com insuficiência venosa crônica

**DOI:** 10.1590/1677-5449.005016

**Published:** 2016

**Authors:** Carlos Alberto Engelhorn, Francisco Eduardo Coral, Isabela Chaves Monteiro Soares, Gabriel Fernando de Araújo Corrêa, Jaqueline Pozzolo Ogeda, Larissa Yuri Hara, Luisa Saemi Murasse

**Affiliations:** 1 Pontifícia Universidade Católica do Paraná – PUC-PR, Curitiba, PR, Brasil.

**Keywords:** varizes, ultrassonografia, refluxo

## Abstract

**Contexto:**

A insuficiência venosa crônica (IVCr) é frequente e predomina nas mulheres, mas ainda há poucas informações sobre o refluxo nas veias safenas na população masculina.

**Objetivos:**

Identificar os diferentes padrões de refluxo nas veias safenas magnas (VSMs) e parvas (VSPs) em homens, correlacionando esses dados com a apresentação clínica conforme a classificação Clínica, Etiológica, Anatômica e Fisiopatológica (CEAP).

**Métodos:**

Foram avaliados 369 membros inferiores de 207 homens pela ultrassonografia vascular (UV) com diagnóstico clínico de IVCr primária. As variáveis analisadas foram a classificação CEAP, o padrão de refluxo nas VSMs e VSPs e a correlação entre os dois.

**Resultados:**

Nos 369 membros avaliados, 72,9% das VSMs apresentaram refluxo com predominância do padrão segmentar (33,8%). Nas VSPs, 16% dos membros inferiores analisados apresentaram refluxo, sendo o mais frequente o padrão distal (33,9%). Dos membros classificados como C4, C5 e C6, 100% apresentaram refluxo na VSM com predominância do refluxo proximal (25,64%), e 38,46% apresentaram refluxo na VSP com equivalência entre os padrões distal e proximal (33,3%). Refluxo na junção safeno-femoral (JSF) foi detectado em 7,1% dos membros nas classes C0 e C1, 35,6% nas classes C2 e C3, e 64,1% nas classes C4 a C6.

**Conclusões:**

O padrão de refluxo segmentar é predominante na VSM, e o padrão de refluxo distal é predominante na VSP. A ocorrência de refluxo na JSF é maior em pacientes com IVCr mais avançada.

## INTRODUÇÃO

A insuficiência venosa crônica (IVCr) é uma afecção bastante comum na população jovem e de meia idade, principalmente em mulheres, sendo sua prevalência progressiva com o aumento da idade^1^.

De acordo com o estudo de Edinburgh, as telangiectasias e as veias reticulares acometem até 85% das mulheres, e as veias varicosas atingem um terço da população de ambos os sexos entre 18 e 64 anos^2^.

Maffei et al. avaliaram 1.755 adultos com mais de 15 anos (443 homens e 1.312 mulheres) e demonstraram uma prevalência de veias varicosas de 47,6%, sendo 37,9% em homens e 50,9% em mulheres^3^.

Nos membros inferiores (MMII), a IVCr manifesta-se com dor, edema, veias varicosas, eczema, hiperpigmentação, atrofia branca, lipodermatoesclerose e úlceras devido à hipertensão venosa causada pelo refluxo nas veias superficiais, perfurantes e/ou profundas. A gravidade clínica da IVCr pode ser avaliada pela classificação Clínica, Etiológica, Anatômica e Fisiopatológica (CEAP)^4^^-^ 6.

De acordo com a classificação CEAP, a clínica da IVCr é classificada em: C0 – sem sinais de doenças venosas visíveis ou palpáveis; C1 – telangiectasias e veias reticulares; C2 – veias varicosas; C3 – presença de edema; C4a – pigmentação parda, eczema; C4b – lipodermatoesclerose ou atrofia branca; C5 – úlcera venosa cicatrizada; e C6 – úlcera venosa ativa^7^.

A ultrassonografia vascular (UV) é o exame de imagem de escolha para avaliar pacientes com IVCr e permite tanto a avaliação anatômica quanto hemodinâmica das veias profundas, safenas e tributárias e veias perfurantes, possibilitando a detecção e localização do refluxo venoso^8^.

Com a identificação e localização anatômica pela UV das fontes de refluxo nas veias safenas magnas (VSMs) e parvas (VSPs) e dos pontos de drenagem de refluxo para o sistema venoso profundo, é possível definir os padrões de refluxo nas veias safenas, o que permite uma avaliação individual de cada extremidade^9^.

Padrões de refluxo venoso foram estudados em pacientes do sexo feminino^8^, mas não existem evidências detalhadas na literatura descrevendo achados especificamente na população masculina.

O objetivo do presente estudo foi identificar os diferentes padrões de refluxo nas veias safenas em pacientes do sexo masculino e correlacionar esses padrões com a apresentação clínica da classificação CEAP.

## MÉTODOS

Foi realizado um estudo transversal em pacientes consecutivos do sexo masculino, com diagnóstico clínico de IVCr e avaliados pela UV.

Foram incluídos pacientes maiores de 18 anos, com IVCr primária e sem cirurgia prévia de varizes. Foram excluídos homens com IVCr secundária e congênita, com tromboflebite recente ou antiga nas veias safenas, e mulheres.

Os pacientes foram avaliados consecutivamente em um período de 4 meses em um laboratório vascular certificado pela ISO 9001 por ultrassonografistas vasculares experientes com certificado de área de atuação pela Sociedade Brasileira de Angiologia e de Cirurgia Vascular (SBACV).

O estudo foi aprovado pelo Comitê de Ética em Pesquisa da Pontifícia Universidade Católica do Paraná (PUC-PR), Curitiba, PR, Brasil, sob protocolo nº 39755314.0.0000.0020.

No momento do estudo pela UV com os pacientes em posição ortostática, foi realizada a avaliação clínica e classificação (CEAP) de cada um dos MMII. Os MMII classificados foram distribuídos em três grupos: C0 ou C1, C2 ou C3, e C4 a C6, correspondendo a IVCr leve, moderada e grave, respectivamente.

### Avaliação ultrassonográfica

Os pacientes foram avaliados com aparelhos Siemens-Antares® e Siemens-X700® (Issaquah, WA, USA), inicialmente para a exclusão de trombose venosa recente ou antiga, em decúbito dorsal, com cortes ultrassonográficos transversais em modo B e manobras de compressibilidade das veias, utilizando transdutores de baixa frequência (5 MHz).

O estudo das VSMs e VSPs foi realizado com os pacientes em posição ortostática, com transdutor de alta frequência (7-10 MHz), para a obtenção das imagens das veias em cortes ultrassonográficos longitudinais em modo B. Com o auxílio do mapeamento em cores do fluxo, foi realizada a pesquisa de refluxo pela manobra de compressão muscular manual distal ao posicionamento do transdutor.

Para a quantificação do refluxo nas veias safenas, foi considerado o tempo de duração do refluxo superior a meio segundo^10^^,^ 11. Não foi considerada a velocidade de pico do refluxo, uma vez que as VSMs não apresentavam tortuosidades ou grandes dilatações. Apesar de não ser o objetivo do trabalho, os diâmetros mensurados variaram entre 6,8 a 9 mm na JSF, 3 a 5,5 mm na coxa e 2,5 a 3,5 mm na perna.

### Padrões de refluxo

**Figura 1:**
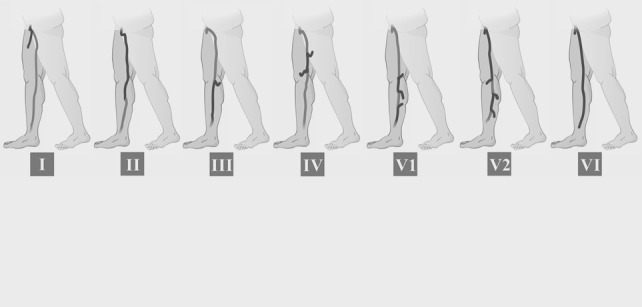
Padrões de refluxo da veia safena magna: **I**) Padrão de refluxo em perijuncional; **II**) Padrão de refluxo proximal; **III**) Padrão de refluxo distal; **IV**) Padrão de refluxo segmentar; **V1**) Padrão de refluxo multissegmentar sem refluxo em JSF; **V2**) Padrão de refluxo multissegmentar com refluxo em JSF; **VI**) Padrão de refluxo difuso.

Os tipos de refluxo (Figuras 1 e 2) nas VSMs e VSPs foram definidos de acordo com a classificação proposta por Engelhorn et al.^9^:

**Figura 2:**
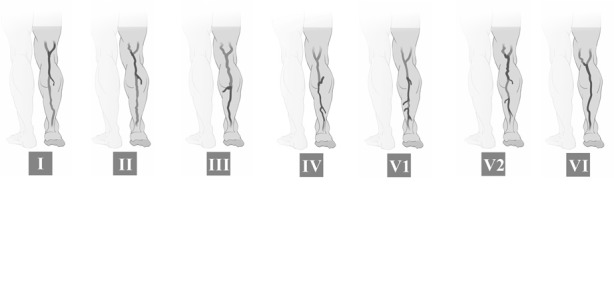
Padrões de refluxo da veia safena parva: **I**) Padrão de refluxo em perijuncional; **II**) Padrão de refluxo proximal; **III**) Padrão de refluxo distal; **IV**) Padrão de refluxo segmentar; **V1**) Padrão de refluxo multissegmentar sem refluxo em JSP; **V2**) Padrão de refluxo multissegmentar com refluxo em JSP; **VI**) Padrão de refluxo difuso.

- Padrão de refluxo perijuncional: caracterizado pelo refluxo na junção safeno-femoral (JSF) ou na junção safeno-poplítea (JSP), escoado por veias tributárias, com manutenção da competência valvular na veia safena.
- Padrão de refluxo proximal: caracterizado por refluxo na JSF ou na JSP, estendendo-se para a veia safena e escoado por veias tributárias superficiais ou veias perfurantes na topografia da coxa ou perna, com manutenção da competência valvular nos segmentos mais distais da veia safena.
- Padrão de refluxo distal: caracterizado pela ausência de refluxo na JSF ou na JSP e na veia safena proximal, com refluxo na veia safena distal até a região perimaleolar causado por veias tributárias superficiais ou veias perfurantes na topografia da coxa ou perna.
- Padrão de refluxo segmentar: caracterizado por um único segmento da veia safena com refluxo na topografia da coxa, do joelho ou da perna, sem envolver a JSF ou a JSP, causado e escoado por veia tributária ou perfurante.
- Padrão de refluxo multissegmentar: caracterizado por dois ou mais segmentos da veia safena com refluxo na topografia da coxa e/ou da perna. Esse padrão de refluxo subdivide-se em multissegmentar com refluxo na JSF ou na JSP e multissegmentar sem refluxo na JSF ou na JSP.
- Padrão de refluxo difuso: caracterizado por refluxo em toda a extensão da veia safena, desde a JSF ou a JSP até a região perimaleolar.

Para avaliação do refluxo na JSF ou JSP, optou-se por agrupar os padrões de refluxo perijuncional, proximal, multissegmentar com refluxo na JSF ou JSP e difuso em um grupo denominado refluxo juncional.

Os resultados das variáveis quantitativas foram descritos por médias, medianas, valores mínimos, valores máximos e desvios padrões. Para variáveis qualitativas foram apresentadas frequências e percentuais. Para avaliação de fatores associados à classificação CEAP, foi considerado o teste qui-quadrado. Valores de p < 0,05 indicaram significância estatística. Os dados foram analisados com o programa computacional IBM SPSS Statistics v20.

## RESULTADOS

Foram avaliados 395 MMII, sendo que 26 membros foram excluídos devido a safenectomia. Foram incluídos dados de 369 MMII de 207 pacientes com idades entre 23 e 85 anos, sendo a média de idade de 48 anos. Dos 207 pacientes, 165 realizaram o exame em ambos os MMII e, em 39, o exame foi realizado unilateralmente, totalizando 184 MMII esquerdos e 185 MMII direitos.

Dos MMII analisados, 108 (29,3%) foram classificados como C0 ou C1; 222 (60,2%) como C2 ou C3; e 39 (10,6%) como C4 a C6.

Com relação à presença de refluxo nas veias safenas, 269 (72,9%) MMII apresentaram refluxo na VSM e 39 (16%) na VSP.

Com relação aos padrões de refluxo nas veias safenas (Tabelas 1 e 2), o refluxo mais frequente na VSM foi o segmentar (33,8%) e na VSP, o refluxo distal (33,9%).

**Tabela 1:** Incidência de refluxo nas veias safenas de acordo com a classificação clínica.

	**C (CEAP** * **)**				
		0 OU 1	2 OU 3	4, 5 OU 6	VALOR DE P
Refluxo na veia safena magna	NÃO	66	34	0	< 0,001
		61,11%	15,32%	0,00%	
	SIM	42	188	39	
		38,89%	84,68%	100,00%	
Refluxo na veia safena parva	NÃO	97	189	24	
		89,81%	85,14%	61,54%	
	SIM	11	33	15	
		10,19%	14,86%	38,46%	

* CEAP: Classificação Clínica, Etiologia, Anatomia e Fisiopatologia.

**Tabela 2:** Incidência dos padrões de refluxo na veia safena magna de acordo com a classificação clínica.

**Padrões de refluxo na veia safena magna**	**CEAP** †		
	0 ou 1 2 ou 3 4, 5 ou 6		
Segmentar	24	63	4
	57,14%	33,51%	10,26%
Proximal	2	24	10
	4,76%	12,77%	25,64%
Multissegmentar com refluxo em JSF	1	30	9
	2,38%	15,96%	23,08%
Multissegmentar sem refluxo em JSF	7	36	5
	16,67%	19,15%	12,82%
Distal	8	22	5
	19,05%	11,70%	12,82%
Difuso	0	13	6
	0,00%	6,91%	15,38%
Total*	42	188	39

* Restrito aos casos que tiveram refluxo na veia safena interna;

† CEAP: Classificação Clínica, Etiologia, Anatomia e Fisiopatologia.

**Figura 3:**
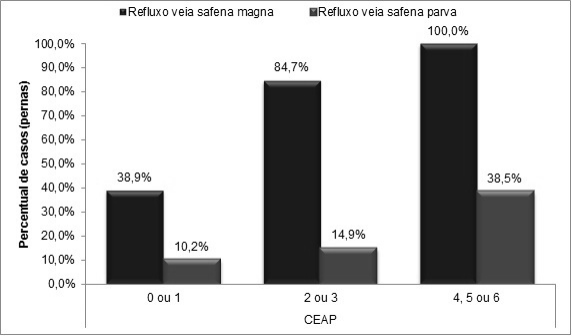
Incidência de refluxo nas veias safenas de acordo com a classificação clínica.

Correlacionando o padrão de refluxo nas veias safenas com a classificação CEAP (Tabela 1), observou-se que nas VSMs cujos membros foram classificados como C4 a C6, 100% apresentaram refluxo. Do mesmo modo, na VSP também houve predominância de refluxo em membros com esses estádios clínicos, sendo esse achado também estatisticamente significativo (Tabela 1 e Figura 3).

Correlacionando os diferentes padrões de refluxo da VSM com a apresentação clínica (Tabela 2), observou-se que nos membros classificados como C0 ou C1 houve predomínio do padrão de refluxo segmentar (57,14%), assim como nos classificados como C2 ou C3 (33,51%). Para as extremidades classificadas como C4, C5 ou C6, a maior incidência foi de refluxos proximal e multissegmentar com refluxo de JSF (25,64% e 23,08%, respectivamente).

Dos 369 MMII avaliados, 95 (25,7%) apresentaram refluxo na JSF. Considerando o refluxo na JSF e correlacionando com as classes clínicas CEAP (Tabela 3), verificou-se uma maior incidência de refluxo (64,1%) nos membros com estádio clínico C4, C5 ou C6 (p < 0,001).

**Tabela 3:** Incidência dos padrões de refluxo na veia safena magna de acordo com a classificação clínica. Os padrões com refluxo em JSF foram agrupados em um único padrão denominado juncional.

**Padrões de refluxo na veia safena magna**	**C (CEAP** **†** **)**		
	**0 ou 1**	**2 ou 3**	**4, 5 ou 6**
Juncional	3	67	25
	7,14%	35,64%	64,10%
Segmentar	24	63	4
	57,14%	33,51%	10,26%
Multissegmentar sem refluxo de junção	7	36	5
	16,67%	19,15%	12,82%
Distal	8	22	5
	19,05%	11,70%	12,82%
Total*	42	188	39

* Valor de p: < 0,001;

† CEAP: Classificação Clínica, Etiologia, Anatomia e Fisiopatologia.

Na VSP, o padrão de refluxo distal foi predominante entre os membros com C0 ou C1 (63,64%). Nos membros com C2 ou C3, os padrões distal, proximal e segmentar se apresentaram com porcentagens muito próximas (24,24%, 27,27% e 30,30%, respectivamente). Para as extremidades C4, C5 ou C6, os padrões distal e proximal foram predominantes, ambos aparecendo em 33,3% dos casos (Tabela 4).

**Tabela 4:** Incidência dos padrões de refluxo na veia safena parva de acordo com a classificação clínica.

**Padrões de refluxo na veia safena parva**	**CEAP** **†**		
	**0 ou 1**	**2 ou 3**	**4, 5 ou 6**
Distal	7	8	5
	63,64%	24,24%	33,33%
Proximal	2	9	5
	18,18%	27,27%	33,33%
Difuso	0	2	1
	0,00%	6,06%	6,67%
Segmentar	0	10	3
	0,00%	30,30%	20,00%
Multissegmentar com refluxo de JSP	0	3	0
	0,00%	9,09%	0,00%
Multissegmentar sem refluxo de JSP	2	1	1
	18,18%	3,03%	6,67%
Total	11	33	15

† CEAP: Classificação Clínica, Etiologia, Anatomia e Fisiopatologia.

## DISCUSSÃO

A pesquisa anatomofuncional pela UV do sistema venoso profundo dos MMII em pacientes com sinais ou sintomas de IVCr permite uma avaliação individualizada de cada extremidade, além de fornecer dados para o planejamento cirúrgico mais adequado e com menor chance de recidiva^8^.

Considerando especificamente a população masculina com IVCr, a literatura é escassa na identificação dos padrões de refluxo nas veias safenas e sua correlação com as diferentes fases da doença.

O presente estudo focou-se justamente nessa população pela análise quantitativa dos refluxos a partir de padrões previamente definidos por Engelhorn et al.^9^, e a correlação com as manifestações clínicas da IVCr.

Nosso trabalho demonstrou a presença de refluxo na maioria (73%) das VSMs e somente em 16% das VSPs, corroborando os achados de um estudo prévio semelhante em mulheres com varizes^12^.

Engelhorn et al.^9^ constataram, em uma população formada por homens e mulheres com IVCr primária, que houve maior incidência na VSM do padrão de refluxo segmentar, seguido pelos padrões multissegmentar sem refluxo de JSF, distal, proximal, multissegmentar com refluxo de JSF e difuso.

Em outro estudo realizado por Engelhorn et al.^12^ em uma população exclusivamente feminina com varizes primárias nos MMII (CEAP 2), também foi demonstrada uma maior incidência do padrão segmentar de refluxo na VSM, seguido pelo padrão multissegmentar sem refluxo de JSF.

Neste trabalho, o padrão de maior incidência também foi o segmentar, seguido pelo multissegmentar sem refluxo de junção, multissegmentar com refluxo de JSF, proximal, distal e difuso. Tais diferenças em relação aos estudos anteriores podem ter ocorrido devido às populações estudadas, o que ressalta a necessidade de avaliar populações específicas. Entretanto, independentemente da população estudada, o refluxo na VSM não é predominante da JSF.

Para a VSP, em nosso estudo, o padrão de refluxo mais frequente foi o distal, seguido pelo proximal e segmentar, demonstrando uma diferença em relação aos padrões identificados nas mulheres (CEAP 2), nas quais houve predomínio do padrão segmentar, seguido dos padrões distal e proximal^12^.

Cassou et al.^13^ identificaram a probabilidade dos diferentes padrões de refluxo em veias safenas de mulheres nos diferentes estádios clínicos da IVCr, sendo que das 288 VSMs nas extremidades identificadas como CEAP C1, 157 (54,51%) não apresentaram refluxo e 87 (30,21%) apresentaram refluxo segmentar. As VSMs das extremidades identificadas como CEAP C2, C3 e C4 apresentaram refluxo segmentar, respectivamente, em 214 (35,97%), 104 (38,10%) e nove (42,86%). Das VSMs das extremidades identificadas como CEAP C5, duas (50%) apresentaram refluxo tipo multissegmentar, e, na CEAP C6, foram encontrados em igual proporção os padrões ausente, segmentar e difuso (33,33%) em cada uma das extremidades.

Do mesmo modo, em nosso trabalho foi correlacionada a apresentação clínica da IVCr com os diferentes padrões de refluxo na VSM. Assim como no trabalho de Cassou et al., nos pacientes do sexo masculino houve maior incidência de refluxo segmentar tanto nas classes C0 e C1 (57%) quanto nas classes C2 ou C3 (33,51%). Esse achado corrobora a hipótese de que a doença venosa se inicia de forma segmentar para posterior degeneração na forma de padrões que abrangem maior extensão na veia.

Por outro lado, na doença venosa mais avançada (C4 a C6) foi identificada a predominância do refluxo juncional em 64% dos MMII. Esse achado concorda com os estudos que demonstraram uma associação entre o acometimento da JSF e formas graves de apresentação clínica da IVCr^14^^,^ 15.

Na VSP, o padrão de refluxo distal foi predominante entre os membros com apresentação clínica C0 ou C1. Já nas classes C2 ou C3, três padrões se destacaram com porcentagens muito próximas de incidência: distal, proximal e segmentar. Em C4, C5 ou C6, os padrões distal e proximal foram os mais incidentes, com o mesmo percentual (33,33%), padrões estes não identificados na população feminina^13^.

Labropoulos et al.^16^ compararam o refluxo venoso e as manifestações clínicas da IVCr em 255 membros inferiores de 217 pacientes sem a definição exata dos diferentes padrões de refluxo, e sim com a atribuição dos refluxos como abrangendo ou não JSF e supra ou infrapatelar. Esses autores encontraram associação entre a ocorrência de refluxo infrapatelar e a presença de sinais clínicos de IVCr mais avançada.

Em nosso trabalho, a ocorrência de refluxo na JSF e JSP, como já apresentado, esteve relacionada a sinais clínicos de IVCr mais avançada (C4 a C6). No entanto, não foi pesquisada a associação do refluxo com a extensão ou localização específica dos refluxos segmentares. Apesar de ser clara a relação encontrada em nosso trabalho entre a presença de refluxo juncional e a maior intensidade das manifestações clínicas de apresentação da doença, talvez, fazendo-se uma análise mais apurada com a diferenciação dos locais de acometimento segmentar e da extensão de cada refluxo, fosse possível comparar nossos achados aos de Labropoulos et al. Assim, seria possível verificar se realmente há relação entre refluxos segmentares abaixo do joelho e a presença de manifestações clínicas mais avançadas^16^.

Conclui-se que o padrão de refluxo segmentar é predominante na VSM, e o padrão de refluxo distal é predominante na VSP. Além disso, as apresentações clínicas iniciais da doença relacionam-se a refluxos segmentares, com maior envolvimento da JSF e da JSP nas apresentações clínicas mais avançadas de IVCr.

## References

[1] Scuderi A, Raskin B, Al Assal F (2002). The incidence of venous disease in Brazil based on the CEAP classification. Int Angiol.

[2] Evans CJ, Fowkes FGR, Ruckley CV, Lee AJ (1999). Prevalence of varicose veins and chronic venous insuficiency in men and women in the general population : Edinburgh Vein Study. J Epidemiol Community Health.

[3] Maffei FH, Magaldi C, Pinho SZ (1986). Varicose veins and chronic venous insufficiency in Brazil: prevalence among 1755 inhabitants of a country town. Int J Epidemiol.

[4] Boisseau MR, Eklof B (2006). Chronic venous disease. N Engl J Med.

[5] Schmid-Schonbein GW, Takase S, Bergan JJ (2001). New advances in the understanding of the pathophysiology of chronic venous insufficiency. Angiology.

[6] Wittens C, Davies AH, Bækgaard N (2015). Management of chronic venous disease. clinical practice guidelines of the European Society for Vascular Surgery (ESVS). Eur J Vasc Endovasc Surg.

[7] Eklof B, Rutherford RB, Bergan JJ (2004). Revision of the CEAP classification for chronic venous disorders: consensus statement. J Vasc Surg.

[8] Coleridge-Smith P, Labropoulos N, Partsch H, Myers K, Nicolaides A, Cavezzi A (2006). Duplex ultrasound investigation of the veins in chronic venous disease of the lower limbs −UIP consensus document. Part I basic principles. Eur J Vasc Endovasc Surg.

[9] Engelhorn CA, Engelhorn AL, Cassou MF, Casagrande C, Gosalan CJ, Ribas E (2004). Classificação anátomo-funcional da insuficiência das veias safenas baseada no eco-Doppler colorido, dirigida para o planejamento da cirurgia de varizes. J Vasc Bras.

[10] van Bemmelen S, Bedford G, Beach K, Strandness DE (1989). Quantitative segmental evaluation of venous valvular reflux with duplex ultrasound scanning. J Vasc Surg.

[11] Labropoulos N, Tiongson J, Pryor L (2003). Definition of venous reflux in lower-extremity veins. J Vasc Surg.

[12] Engelhorn CA, Engelhorn AL, Cassou MF, Salles-Cunha SX (2005). Patterns of saphenous reflux in women with primary varicose veins. J Vasc Surg.

[13] Cassou MF, Gonçalves PCZ, Engelhorn CA (2007). Probabilidade de refluxo nas veias safenas de mulheres com diferentes graus de insuficiência venosa crônica. J Vasc Bras.

[14] Garcia-Gimeno M, Rodriguez-Camarero S, Tagarro-Villalba S (2013). Reflux patterns and risk factors of primary varicose veins’ clinical severity. Phlebol J Venous Dis..

[15] Pittaluga P, Chastanet S, Rea B, Barbe R (2008). Classification of saphenous refluxes: implications for treatment. Phlebol J Venous Dis..

[16] Labropoulos N, Leon M, Nicolaides AN, Giannoukas AD, Volteas N, Chan P (1994). Superficial venous insufficiency: correlation of anatomic extent of reflux with clinical symptoms and signs. J Vasc Surg.

