# Supplementary material for: Padrões de refluxo nas veias safenas em homens com insuficiência venosa crônica
Source: J Vasc Bras. 2016 Oct-Dec;15(4):268–74. [Article in Portuguese] doi: 10.1590/1677-5449.005016 (PMC5829724; doi:10.1590/1677-5449.005016)
Supplement: Translation

# Translation: Patterns of saphenous reflux in men with chronic venous insufficiency

**Authors:** Carlos Alberto Engelhorn, Francisco Eduardo Coral, Isabela Chaves Monteiro Soares, Gabriel Fernando de Araújo Corrêa, Jaqueline Pozzolo Ogeda, Larissa Yuri Hara, Luisa Saemi Murasse

**Affiliations:** 1 Pontifícia Universidade Católica do Paraná – PUC-PR, Curitiba, PR, Brazil.

**Keywords:** varicose veins, ultrasonography, reflux

## Abstract

**Background:**

Chronic venous insufficiency (CVI) is frequent and predominantly affects women, but there is a lack of information about saphenous vein reflux in the male population.

**Objective:**

To identify different patterns of reflux in the great and small saphenous veins of men and correlate them with clinical presentation graded according to the Clinical-Etiology-Anatomy-Pathophysiology (CEAP) classification.

**Methods:**

A total of 369 lower limbs in 207 men with a clinical diagnosis of primary CVI of the lower limbs were evaluated using vascular ultrasound (VU). The variables analyzed were CEAP clinical classification, patterns of reflux in the great and small saphenous veins, and the correlations between them.

**Results:**

A total of 369 limbs were evaluated and in 72.9% of them the great saphenous vein (GSV) had reflux, predominantly the segmental pattern (33.8%), while in 16% of the lower limbs analyzed the small saphenous vein (SSV) had reflux, among which the most frequent pattern was distal (33.9%). All limbs classified as C4, C5, or C6 had GSV reflux, predominantly proximal (25.64%), while 38.46% had SSV reflux compatible with distal and proximal reflux patterns (33.3%). Reflux was detected at the saphenofemoral junction (SFJ) in 7.1% of limbs graded C0 and C1, in 35.6% of C2 and C3 limbs and in 64.1% of C4 to C6 limbs.

**Conclusion:**

The predominant reflux patterns are segmental at the GSV and distal at the SSV. The frequency of SFJ reflux is higher in patients with more advanced CVI.

## INTRODUCTION

Chronic venous insufficiency (CVI) is a very common condition in the young and middle-aged population, primarily in women, and its prevalence increases progressively with age.^1^

According to the Edinburgh study, telangiectasias and reticular veins affect up to 85% of women, while one third of the population of both sexes aged 18 to 64 years have varicose veins.^2^

Maffei et al. assessed 1,755 adults over the age of 15 years (443 men and 1,312 women) and demonstrated a 47.6% prevalence of varicose veins: 37.9% in men and 50.9% in women.^3^

In the lower limbs (LL), CVI manifests with pain, edema, varicose veins, eczema, hyperpigmentation, athrophie blanche, lipodermatosclerosis, and ulcers resulting from venous hypertension caused by reflux in superficial, perforating and/or deep veins. The clinical severity of CVI can be graded according to the Clinical-Etiology-Anatomy-Pathophysiology classification (CEAP).^4^^-^ 6

According to the CEAP classification, CVI clinical status is graded as follows: C0 – no visible or palpable signs of venous disease; C1 – telangiectasias and reticular veins; C2 – varicose veins; C3 –edema; C4a – pigmentation or eczema; C4b – lipodermatosclerosis or athrophie blanche; C5 – healed venous ulcer; or C6 – active venous ulcer.^7^

Vascular ultrasonography (VU) is the imaging exam of choice for evaluation of patients with CVI, enabling anatomic and hemodynamic assessment of the deep, saphenous, tributary, and perforating veins, and detection and location of venous reflux.^8^

Once sources of reflux in the great saphenous veins (GSVs) and small saphenous veins (SSVs) and the points at which the reflux drains into the deep vein system have been identified and located anatomically using VU, it is possible to define the patterns of saphenous vein reflux, offering an individual assessment of each extremity.^9^

Venous reflux patterns have been studied in female patients,^8^ but there is no detailed evidence in the literature describing findings specific to the male population.

The objective of the present study was to identify the different patterns of reflux in the saphenous veins of male patients and to correlate these patterns with clinical presentation graded according to the CEAP classification.

## METHODS

A cross-sectional study based on clinical diagnoses of CVI and VU assessments was conducted with a consecutive series of male patients.

Inclusion criteria were male patients over the age of 18 years, with primary CVI and no previous varicose vein surgery. Exclusion criteria were secondary and congenital CVI, recent or long-term thrombophlebitis of the saphenous veins, and being female.

Patients were assessed consecutively over a 4-month period at a vascular laboratory with ISO 9001 certification by experienced vascular ultrasonographers with certification from the Brazilian Society for Angiology and Vascular Surgery (Sociedade Brasileira de Angiologia e de Cirurgia Vascular).

The study was approved by the Research Ethics Committee at the Pontifícia Universidade Católica do Paraná (PUC-PR), Curitiba, PR, Brazil, under protocol number 39755314.0.0000.0020.

Patients were examined with VU while standing upright and at the same time a clinical assessment was made of each LL, which were then classified using the CEAP. Each LL was allocated to one of three groups on the basis of CEAP grade: C0 and C1, C2 and C3, or C4 to C6, equivalent to mild, moderate and severe CVI respectively.

### Ultrasonographic assessment

Patients were examined in decubitus dorsal using Siemens-Antares® and Siemens-X700® equipment (Issaquah, WA, USA), initially to rule out recent or past venous thrombosis, using transverse ultrasound slices in B mode and vein compressibility maneuvers with a low frequency transducer (5 MHz).

Great and small saphenous veins were examined with the patients standing upright, using a high frequency transducer (7-10 MHz) to acquire images of the veins on longitudinal ultrasound slices in B mode. With the aid of color flow mapping, manual compression maneuvers distal of the transducer were used to screen for reflux.

Quantification of reflux in the saphenous veins was based on a duration of reflux greater than half a second.^10^^,^ 11 Peak reflux velocity was not taken into account because the GSVs did not exhibit tortuosity or major dilations. Although unrelated to the study objective, diameters were measured and found to vary from 6.8 to 9 mm at the SFJ, 3 to 5.5 mm in the thigh, and 2.5 to 3.5 mm in the leg.

### Reflux patterns

The types of reflux (Figures 1 and 2) observed in GSVs and SSVs were defined according to the classification proposed by Engelhorn et al.,^9^ as follows:

**Figure 1:** Patterns of reflux in the great saphenous vein: **I**) Medial junction reflux pattern; **II**) Proximal reflux pattern; **III**) Distal reflux pattern; **IV**) Segmental reflux pattern; **V1**) Multisegmental pattern without reflux at SFJ; **V2**) Multisegmental pattern with reflux at SFJ; **VI**) Diffuse reflux pattern.

**Figure 2:** Patterns of reflux in the small saphenous vein: **I**) Medial junction reflux pattern; **II**) Proximal reflux pattern; **III**) Distal reflux pattern; **IV**) Segmental reflux pattern; **V1**) Multisegmental pattern without reflux at SPJ; **V2**) Multisegmental pattern with reflux at SPJ; **VI**) Diffuse reflux pattern.

- Perijunctional reflux pattern - characterized by reflux at the saphenofemoral junction (SFJ) or saphenopopliteal junction (SPJ) drained by tributaries, with valvular competence of the saphenous vein.
- Proximal reflux pattern - characterized by reflux at the SFJ or SPJ, extending to the saphenous vein, drained by superficial tributary or perforating vein at thigh or leg level, with preservation of valvular competence in more distal segments of the saphenous vein.
- Distal reflux pattern - characterized by absence of reflux in SFJ and SPJ and in the proximal saphenous vein, with reflux in the great saphenous vein up to the medial malleolus, caused by superficial tributary or perforating vein at thigh or leg level.
- Segmental reflux pattern - characterized by reflux in a single segment of the great saphenous vein at thigh, knee and/or leg level, with no involvement of SFJ or SPJ, caused and drained by a tributary or perforating vein.
- Multisegmental reflux pattern - characterized by reflux in two or more segments of the great saphenous vein at thigh and/or leg level. This reflux pattern is divided into multisegmental with reflux at SFJ or SPJ and multisegmental without reflux at SFJ or SPJ.
- Diffuse reflux pattern - characterized by reflux in the whole of the great saphenous vein, from the saphenofemoral junction up to the medial malleolus.

For the purposes of assessing reflux at the SFJ or SPJ, the perijunctional reflux, proximal reflux, multisegmental with reflux at SFJ or SPJ and diffuse reflux patterns were all grouped together in a “junctional” reflux pattern.

Results for quantitative variables were expressed as means, medians, minimum and maximum values, and standard deviations. Qualitative variables were expressed as frequencies and percentages. The chi-square test was used to analyze factors associated with CEAP classifications. Results with p values < 0.05 were considered statistically significant. Data were analyzed using IBM SPSS Statistics v20.

## RESULTS

A total of 395 LL were evaluated, but 26 limbs were excluded because of saphenectomies. The study sample therefore comprised 369 LL from 207 patients aged 23 to 85 years, with a mean age of 48 years. Of these 207 patients, 165 had both LL examined and 39 had a unilateral examination, resulting in 184 left LL and 185 right LL.

Clinical assessment of the LL resulted in 108 (29.3%) LL classified as C0 or C1; 222 (60.2%) as C2 or C3; and 39 (10.6%) as C4, C5 or C6.

With regard to presence of reflux in the saphenous veins, 269 (72.9%) LL exhibited reflux in the GSV and 39 (16%) in the SSV.

The results for reflux patterns in the saphenous veins (Tables 1 and 2) showed that the most common type of reflux in the GSV was the segmental reflux pattern (33.8%) and the most common type in the SSV was the distal reflux pattern (33.9%).

**Table 1:** Incidence of reflux in the saphenous veins, by clinical classification.

	**C (CEAP** * **)**				
		0 OR 1	2 OR 3	4, 5 OR 6	P VALUE
Reflux in the great saphenous vein	NO	66	34	0	< 0.001
		61.11%	15.32%	0.00%	
	YES	42	188	39	
		38.89%	84.68%	100.00%	
Reflux in the small saphenous vein	NO	97	189	24	
		89.81%	85.14%	61.54%	
	YES	11	33	15	
		10.19%	14.86%	38.46%	

* CEAP: Clinical-Etiology-Anatomy-Pathophysiology classification.

**Table 2:** Incidence of patterns of reflux in the great saphenous vein by clinical classification.

**Reflux patterns in the great saphenous vein**	**CEAP** †		
	0 or 1 2 or 3 4, 5 or 6		
Segmental	24	63	4
	57.14%	33.51%	10.26%
Proximal	2	24	10
	4.76%	12.77%	25.64%
Multisegmental with reflux at SFJ	1	30	9
	2.38%	15.96%	23.08%
Multisegmental without reflux at SFJ	7	36	5
	16.67%	19.15%	12.82%
Distal	8	22	5
	19.05%	11.70%	12.82%
Diffuse	0	13	6
	0.00%	6.91%	15.38%
Total*	42	188	39

* Restricted to cases with reflux in the internal saphenous vein;

† CEAP: Clinical-Etiology-Anatomy-Pathophysiology classification.

Correlating the reflux patterns in saphenous veins with the CEAP classifications (Table 1) showed that 100% of GSVs in limbs classified as C4 to C6 exhibited reflux. In a similar manner, there was also a predominance of reflux in the SSVs of limbs with these clinical grades and this finding was also statistically significant (Table 1 and Figure 3).

**Figure 3:** Incidence of reflux in the saphenous veins, by clinical classification.

Correlating the different reflux patterns in GSVs with clinical presentation (Table 2) showed that the segmental reflux pattern was the most common in limbs classified as C0 or C1 (57.14%) and also in limbs classified as C2 or C3 (33.51%). However, the highest incidence rates in extremities classified as C4, C5, or C6 were for proximal reflux and multisegmental reflux with SFJ reflux (25.64% and 23.08% respectively).

Ninety-five of the 369 LL assessed exhibited reflux at the SFJ (25.7%). Correlation of reflux at the SFJ with CEAP clinical grades (Table 3) showed that there was a higher incidence of reflux (64.1%) in limbs with C4, C5, or C6 clinical status (p < 0.001).

**Table 3:** Incidence of reflux patterns in the great saphenous vein, by clinical classification. Patterns including reflux in the SFJ were combined into a single group entitled junctional reflux pattern.

**Reflux patterns in the great saphenous vein**	**C (CEAP** **†** **)**		
	**0 or 1**	**2 or 3**	**4, 5 or 6**
Junctional	3	67	25
	7.14%	35.64%	64.10%
Segmental	24	63	4
	57.14%	33.51%	10.26%
Multisegmental without reflux in junctions	7	36	5
	16.67%	19.15%	12.82%
Distal	8	22	5
	19.05%	11.70%	12.82%
Total*	42	188	39

* P value: < 0.001;

† CEAP: Clinical-Etiology-Anatomy-Pathophysiology classification.

In the SSV, the distal reflux pattern predominated in limbs graded C0 or C1 (63.64%). In limbs classed as C2 or C3, the distal, proximal, and segmental patterns all had very similar percentages (24.24%, 27.27%, and 30.30%, respectively). In C4, C5, and C6 limbs, the distal and proximal patterns predominated, both seen in 33.3% of cases (Table 4).

**Table 4:** Incidence of reflux patterns in the small saphenous vein, by clinical classification.

**Reflux patterns in the small saphenous vein**	**CEAP** **†**		
	**0 or 1**	**2 or 3**	**4, 5 or 6**
Distal	7	8	5
	63.64%	24.24%	33.33%
Proximal	2	9	5
	18.18%	27.27%	33.33%
Diffuse	0	2	1
	0.00%	6.06%	6.67%
Segmental	0	10	3
	0.00%	30.30%	20.00%
Multisegmental with reflux in the SPJ	0	3	0
	0.00%	9.09%	0.00%
Multisegmental without reflux in the SPJ	2	1	1
	18.18%	3.03%	6.67%
Total	11	33	15

† CEAP: Clinical-Etiology-Anatomy-Pathophysiology classification.

## DISCUSSION

Using VU to conduct anatomic and functional assessment of the deep vein system in the LL of patients with signs or symptoms of CVI offers the possibility of conducting an individualized evaluation of each extremity and provides data to improve planning for surgery, reducing the recurrence of varicose veins.^8^

Specifically with regard to the male population with CVI, there is scant literature identifying reflux patterns in saphenous veins and their correlations with different phases of the disease.

This study investigated this population using quantitative analysis of reflux types based on patterns defined by Engelhorn et al.,^9^ and analyzed their correlations with the clinical manifestations of CVI.

Our results demonstrate that reflux was present in the majority (73%) of GSVs, but in just 16% of the SSVs, confirming the findings of a similar study conducted previously with women with varicose veins.^12^

Engelhorn et al.^9^ studied a population made up of men and women with primary CVI and found that the highest incidence rate in the GSV was for the segmental reflux pattern, followed by multisegmental without reflux at the SFJ, distal reflux, proximal, multisegmental with reflux at the SFJ, and the diffuse reflux pattern.

In another study by Engelhorn et al.,^12^ conducted with an entirely female population with primary varicose veins in the LL (CEAP 2), it was also found that the segmental reflux pattern had the highest incidence in the GSV, followed by the multisegmental pattern without reflux at the SFJ.

In this study, the pattern with highest incidence was also the segmental, followed by multisegmental without junctional reflux, multisegmental with reflux at the SFJ, proximal reflux, distal reflux, and the diffuse reflux pattern. These differences in relation to previous studies may have been because of the populations studied, which underscores the need to assess specific populations. However, irrespective of study population, in the GSV, patterns in which reflux is not present at the SFJ predominate.

In our study, the most common reflux pattern in the SSV was the distal type, followed by proximal reflux and segmental reflux, demonstrating a difference in comparison to the patterns identified in women (CEAP 2), among whom the segmental reflux pattern predominated, followed by the distal and proximal reflux patterns.^12^

Cassou et al.^13^ identified the probability of different reflux patterns in the saphenous veins of women at different clinical stages of CVI, finding that 157 out of 288 GSVs (54.51%) in extremities classified as CEAP C1 did not have reflux, while 87 (30.21%) of them had segmental reflux. In turn, 214 (35.97%), 104 (38.10%) and nine (42.86%) GSVs in extremities classified as CEAP C2, C3, and C4 respectively had segmental reflux. Two (50%) GSVs in extremities classified as CEAP C5 exhibited multisegmental reflux and in CEAP C6 limbs the same proportion of absent, segmental, and diffuse patterns (33.33%) were detected in all extremities.

In our study, patients’ CVI clinical presentations were correlated with different GSV reflux patterns. In common with a study by Cassou et al., among the male patients there was a greater incidence of segmental reflux in classes C0 and C1 (57%) and in classes C2 and C3 (33.51%). This finding supports the hypothesis that venous disease begins in a segmental form and later degenerates into patterns that involve more segments of the vein.

In contrast, predominance of junctional reflux was observed in 64% of LL with more advanced venous disease (C4 to C6). This finding is in agreement with studies that have demonstrated an association between SFJ involvement and severe forms of CVI clinical presentation.^14^^,^ 15

In the SSV, the distal reflux pattern predominated in limbs with C0 or C1 clinical presentation. In classes C2 and C3, three different patterns, the distal, proximal, and segmental patterns, all had very similar percentages of incidence. In C4, C5, and C6 limbs the distal and proximal patterns had the highest incidence rates, all with the same percentage (33.33%), and these patterns were not observed in the female population.^13^

Labropoulos et al.^16^ compared venous reflux and clinical manifestations of CVI in 255 lower limbs from 217 patients and while they did not use exact definitions of different reflux patterns, they did report whether or not reflux involved the SFJ and labeled reflux as suprapatellar or infrapatellar. These authors found an association between occurrence of infrapatellar reflux and presence of clinical signs of more advanced CVI.

In our study, as already explained, occurrence of reflux in the SFJ and SPJ was related to clinical signs of more advanced CVI (C4 to C6). However, the possibility of associations between reflux and the extent or specific location of segmental reflux was not analyzed. While there was a clear relationship in our study between junctional reflux and greater intensity of clinical manifestations of disease presentation, it is possible that if a more detailed analysis had been conducted, including differentiation of the sites of involvement in segmental patterns and the extent of each reflux, it would have been possible to compare our findings with those reported by Labropoulos et al. It would then be possible to determine whether there really is a relationship between segmental reflux below the knee and the presence of more advanced clinical manifestations.^16^

It can be concluded that the segmental reflux pattern predominates in the GSV and the distal reflux pattern predominates in the SSV. Additionally, the initial clinical presentations of the disease are related to segmental reflux patterns, with greater SFJ and SPJ involvement in more advanced CVI clinical presentations.
